# Ritual Circumcision Followed by Gangrene of the Penis with a Fatal Termination

**Published:** 1898-09

**Authors:** 


### RITUAL CIRCUMCISION FOLLOWED BY GANGRENE OF THE PENIS WITH A FATAL TERMINATION.

Dr. A. Brothers (*Medical Record*) reports the following case: The little victim was, as is required by the Jewish law, circumcised on the eighth day of his earthly existence, by one of those ignorant religious operators called "Mohels." On the night of the circumcision the child began to bleed profusely and the "Mohel" was sent for. After working on the child for four hours, he succeeded in stopping the hemorrhage—how he stopped it will be seen presently. The child became feverish, vomited, and fell into a condition of collapse. The doctor was called in and found the following condition: The abdomen protruded on account of a distended bladder, which extended half way up to the umbilicus. The penis was almost entirely denuded of skin and presented a gangrenous, black, hard mass, about three-quarters of an inch in length. On examination, the doctor discovered a narrow strip of gauze constricting the gangrenous portion of the penis. After removing the strip of gauze with difficulty, and cutting away a portion of the charcoal-like tissue, the uretha was reached and free discharge of urine took place. The child later developed convulsions, abscesses, vomited blood, and died from exhaustion a month after the circumcision.

